# MicroRNA-153 promotes brain-derived neurotrophic factor and hippocampal neuron proliferation to alleviate autism symptoms through inhibition of JAK-STAT pathway by LEPR

**DOI:** 10.1042/BSR20181904

**Published:** 2019-06-25

**Authors:** Yu-Hui You, Zhi-Qiang Qin, Huan-Li Zhang, Zhao-Hong Yuan, Xin Yu

**Affiliations:** Department of Paediatric Rehabilitation, Affiliated Hospital of Jining Medical University, Jining 272029, P.R. China

**Keywords:** Autism, Brain-derived neurotrophic factor, Hippocampal neurons, JAK-STAT signaling pathway, microRNA-153, Proliferation

## Abstract

Autism is known as a severe neurobehavioral syndrome, with males affected more often than females. Previous studies have revealed that microRNAs (miRNAs) play a critical role in the search for novel therapeutic strategies for autism. Therefore, we evaluate the ability of miR-153 to influence brain-derived neurotrophic factor (BDNF) of autism as well as proliferation and apoptosis of hippocampal neuron through the janus kinase-signal transducer and activator of transcription (JAK-STAT) signaling pathway by targeting leptin receptor (LEPR). Firstly, the autistic mice models were established and Morris water maze was employed for the analysis of the learning ability and memory of the mice. Besides, in vitro experiments were conducted with the transfection of different mimic, inhibitor, or siRNA into the hippocampal neuron cells, after which the effect of miR-153 on LEPR and the JAK-STAT signaling pathway-related factors was investigated. Next, 3-[4,5-dimethylthiazol-2-yl]-2,5 diphenyl tetrazolium bromide assay and flow cytometry assay were conducted to evaluate cell proliferation, cell cycle, and apoptosis respectively following transfection. The results revealed that there was a significant decrease in learning ability and memory in the autistic mice along with a reduction in the positive expression rate of BDNF and serious inflammatory reaction. LEPR was confirmed as a target gene of miR-153 by the dual luciferase reporter gene assay. After transfection of overexpressed miR-153, LEPR and the JAK-STAT signaling pathway were inhibited followed by an increase in BDNF and enhancement of cell proliferation. In conclusion, the high expression of miR-153 can inhibit activation of JAK-STAT signaling pathway by LEPR, thus improving BDNF expression and the proliferative ability of hippocampal neurons.

## Introduction

Autism, a kind of life-long neurodevelopmental disability, is often accompanied with people’s deficiencies in social interaction, communication, and repeated and stereotyped behaviors [1]. Autism affects males more than females, due to the wide-ranging influence of fetal testosterone, a prominent sex steroid produced in males, on phenotypic variability involving autism [2]. Autism is usually diagnosed in early childhood, and the median prevalence of autism is 0.62% [3,4]. The occurrence of autism has been linked to a number of genetic and environmental factors, and some of the widely recognized risk factors include certain infections during pregnancy such as rubella, and the use of alcohol, valproic acid, or cocaine during pregnancy [5,6]. The existing regimen for autism therapy includes psychoeducation, coordination of care, and modified cognitive behavioral therapy [7]. There’s been an increasing number of researches conducted with the chief focus of finding new strategies to the treatment of autism due to the inefficacy of the currently used treatment. The use of microRNAs (miRNAs) as biomarkers has been of particular importance in improving the diagnosis, prognosis, and treatment of autism [8–10].

MiRNAs, a class of small non-coding RNAs regulating gene expression and neurodevelopment, are considered as potential targets in the development of novel therapeutic strategies [11–13]. MiR-153, an intronic miRNA recognized as a modulator of alpha-synuclein at post-transcription level, has been identified to be a significant component of the brain with an example that reflects synuclein expression in different tissues in the period of neuronal development, indicating that they take coordinated effects in alpha-synuclein [14]. Leptin receptor (LEPR) was found to be a target gene of miR-153 based on the results from the online website at (microRNA.org). Located at 1p31, LEPR is a pleiotropic molecule which appears in a variety of tissues and affects certain cellular functions, and acts as an inflammatory cytokine, hence influencing the pathogenesis of multiple tumors [15–18]. With the utilization of janus kinase-signal transducer and activator of transcription (JAK-STAT) inhibitors, the JAK-STAT signaling pathway regulates the innate immune response and improves neuroinflammation, which is essential for the development and function of both innate and adaptive immunity [19]. Besides, JAK-STAT signaling pathway plays an essential role in immune dysfunction in autism as autistic patients can relieve from inhibition of JAK-STAT signaling [20]. In recent days, there’s been an increase in research demonstrating the role of miRNA in autism pathogenesis; however, there is lack of information regarding the correlation between miR-153 and autism. The present study was conducted to explore the effect of miR-153 alternations on autism involving the JAK-STAT signaling pathway.

## Materials and methods

### Model establishment

Thirty healthy adult C57BL/6J mice (The Animal Experiment Center of Dalian Medical University) at clean level, including 15 male mice (weight of 26–28 g) and 15 female mice (weight of 25–27 g), were used in the present study. They were fed in the special-purpose animal room of Affiliated Hospital of Jining Medical University, with free access to food and water. The mice were ensured 12 h of illumination (7:00–19:00 for each day) with quiet environment at 25 °C. Experimental animal feeding and experimental procedures were strictly in accordance with the experimental animal protection provisions.

After an adaptive phase that lasted for 2 weeks, the male and female mice were equally divided in pairs into 15 cages overnight. The following morning, vaginal smears were obtained and evaluated from the female mice. The date at which sperm was detected was considered as the indicator of pregnancy and recorded as 0.5 gestational age, after which the female mice were fed separately. According to the random digital table method, the pregnant mice were divided into two groups with eight mice in each group. On the 12.5th day of pregnancy, the female mice received intraperitoneal injection of sodium valproate (VPA; 600 mg/kg, Sigma Bio Technology Co., Ltd., California, U.S.A.), which had been diluted with 0.85% of normal saline to 250 mg/ml solution, and the births of mice were labeled as the VPA group. Meanwhile in the remaining group, the male and female mice mated normally, after which seven pregnant mice were obtained which then received the same treatment with normal saline, and births of mice were labeled as the normal saline group (the NS group). All female mice were able to conceive successfully, and each of them gave birth to 5–12 baby mice. Afterwards, 40 mice were separately selected from the VPA group (70 mice) and the NS group (65 mice) to take part in the developmental behavioral assessment [21]. Their birth date was recorded as the first day after birth (P1).

### Auxology measurement

Ten mice with age of P7, P14, P21, P56, and P90 were separately selected from the VPA group and the NS group and weighed to analyze the average weight (g) of all ages. Eyes open (EO) duration measurement: 10 mice were selected from the VPA group and the NS group with the age of P12, P13, P14, P15, and P16 to observe the EO condition, and the score criteria was as follows: neither eyes were open (0 point), only one eye was open (1 point), and both two eyes were open (2 points). Turning tendency detection-hang plate test: 10 mice were selected from the age of P7, P8, P9, and P10 between the VPA group and the NS group, to observe the mice’s directional trend. The mice were placed upside down on the smooth inclined plane with 25 angles to observe their ability to turn to 180 angles and the average time (s) it took to complete the turning. The turning time was an indication of the mice’s vestibular sensations and physiogeny. Coordination detection – swimming test: 10 mice were separately chosen from the VPA and NS groups with age of P9, P11, P13, and P15, and were placed at the centre of a thermostatic container and observed for 5–10 s, and the score criteria was as follows: the top of head and nose were under the water (0 point), the nose was below the water while the top of head was above the water (1 point), both the head and the nose were above the water or at the water level with the ears under the water (2 points), the nose and the head were beyond the water with the water level at the middle part of the ears (3 points), and the nose and the head were beyond the water with the water level beneath the ears (4 points).

### Morris water maze (MWM) navigation experiment

This step included a series of experiments including space exploration, the reverse, visual platform, and hidden platform. The detection system consisted of a mobile transparent platform, an automatic recording system and a round pool. The pool was 120 cm in diameter, 50 cm in height, and 30 cm in depth (2 cm higher than the platform). The temperature of water was adjusted to that of room temperature. The platform was put into the first quadrant, and two entry water points were selected on the opposite side of the platform that was the same distance as the platform. The mice were allowed to acclimatize the environment 1 day prior to the experiment. The position of platform was fixed when conducting experiment. When the mice were trained, they were slowly placed in water from three starting points (with the exception of the fourth quadrant) with the mice facing the wall of the pool. Meanwhile, the recording device was started up with the escape latency calculated, and then mice were left to stay at the platform for 10 s. Provided that the mice were not able to find the platform after 60 s, the escape latency was recorded as 60 s, after which the next water entry point training was started. The first practice was made with the mice entering the water pool separately from three different water entry points. The average latent period of the three quadrants was set as the escape latency of this navigation experiment, and the escape latency was applied to determine the space learning and memory ability. The shorter the escape latency was the better their space learning and memory ability were. All mice participated in the above experiments. Three days later, the escape latency was recorded twice a day for a consecutive 5 days. Day escape latency was made according to the average escape latent period of the day. The duration of the escape latency was used to ensure the changes of mice memory capability and to preliminarily assess the establishment of the mice models.

### Immunohistochemistry (IHC)

Fifteen mice were selected from each group and received intraperitoneal injection with 20 mg/kg 1% sodium pentobarbital. Once the anesthesia was administered, the thoracic cavity of mice was excised. The mice were then intubated through the left ventricle to aorta, and irrigated with normal saline, added with 100 ml phosphate buffer solution (PBS), and were fixed with 100 ml 4% paraformaldehyde. Following the removal of the head, the brain was obtained immediately. The samples were then fixed with formaldehyde, embedded by paraffin, and sliced into 4-μm serial sections. The temporal lobe cerebral cortex brain tissue sections were placed into a 60°C incubator for 1 h, dewaxed with three jars of xylene (each for 10 min), and dehydrated by gradient alcohol of 95, 80, 75% (each for 1 min). After receiving a wash with running water for 1 min, the sections were incubated with 3% hydrogen peroxide (84885, sigma, San Francisco, California, U.S.A.) at 37°C for 30 min. After being rinsed with PBS for 3 min, the sections were boiled in 0.01 M citric acid buffer at 95°C for 20 min, cooled to room temperature, and washed by PBS. The sections were then blocked by normal goat serum fluid at 37°C for 10 min, incubated with the primary antibody rabbit anti-mouse LEPR (1 : 20 to 1 : 200, ab222823, Abcam, Cambridge, U.S.A.) and brain-derived neurotrophic factor (BDNF) (1 : 200, ab108319, Abcam, Cambridge, U.S.A.) overnight at 4°C and then washed by PBS. Subsequently, the sections were incubated with the horseradish enzyme labeled secondary antibody goat anti-rabbit (DF7852, Shanghai Yun Yao Bio Technology Co., Ltd., Shanghai, China) for 30 min, treated with streptomycin anti-biotin-peroxidase complex for 30 min, developed by diaminobenzidine (DAB) (DA1010-3mL, Beijing Solarbio Science & Technology Co., Ltd., Beijing, China) for 5–10 min, re-stained with hematoxylin and mounted. The primary antibody was then replaced by PBS which was used as the negative control. Five high-power fields (HPF) (400×) were randomly selected with 100 cells in each field in order to count the number of positive cells of total field cell percentage, and positive cells/total cells > 10% was considered positive (+), and positive cells ≤ 10% was considered negative (−). The experiment was repeated three times.

### Terminal deoxynucleotidyl transferase-mediated dUTP nick end labeling staining

TUNEL kit (Roche, Basel, Switzerland) was used to stain and observe cell apoptosis in temporal lobe cerebral cortex brain tissue of mice. Paraffin-embedded sections were dewaxed by xylene, and rehydrated with gradient ethanol. The sections were then washed with PBS for 5 min, added with 50 μl protease K (20 μg/ml, P6556, Sigma Bio Technology Co., Ltd., California, U.S.A.) solution, and then hydrolyzed at room temperature for 20 min to remove tissue protein. Next, the sections were washed with PBS three times (each time for 5 min), and added with citrate for antigen repair for 30 min. In accordance with the instructions on the TUNEL detection kit, the sections were added with 50 μl TUNEL reaction solution (enzyme solution : marking liquid = 1 : 9) for reaction for 50 min, and washed with PBS three times (each time for 5 min). After being dried, the sections were incubated with 50 μl transition agent-peroxidase (POD) at 37°C for 30 min, and developed by DAB (Beijing Solarbio Science & Technology Co., Ltd., Beijing, China) for 3 min. After being washed with PBS three times (each time for 5 min) and dried, the sections were re-stained with hematoxylin for 3 s and mounted by neutral balsam. Finally, high power fields under the microscope were randomly selected to count the apoptotic neurons, and the apoptosis index (AI) = the number of apoptotic cells/total cells × 100%.

### Enzyme-linked immunoassay (ELISA)

ELISA kits (Shanghai Enzyme-linked Biotechnology Co., Ltd. Shanghai, China) were performed to detect interleukin-6 (IL-6), IL-1β and tumor necrosis factor alpha (TNF-α). IL-6 (ab100713, Abcam, Cambridge, U.K.), IL-1β (ab46052, Abcam, Cambridge, U.K.), and TNF-α (ab34674, Abcam, Cambridge, U.K.) were diluted by antibodies to 1–10 μg/ml, with 0.1 ml added to each well for incubation at 4°C overnight, after which the samples were washed three times the following day. The above reaction wells were added with certain amount of dilute supernatant (0.1 ml) and incubation was carried out at 37°C for 1 h and washed. Simultaneously, the blank, negative, and positive control wells were prepared in the reaction well, and 0.1 ml freshly diluted enzyme labeled secondary antibody (Abcam Inc., Cambridge, MA, U.S.A.) was added for incubation at 37°C for 35–40 min and washed, followed by washing with ddH_2_O (PER 018-1, Beijing Dingguo Changsheng Biotechnology Co., Ltd., Beijing, China). Each reaction well was added with temporarily prepared TMB substrate solution (EL0001, InnoReagents, Zhejiang, China) at 37°C for 10–30 min, and 50 μl stop buffer was added to terminate the development. Finally, the optical density (OD) of each well was determined at the wavelength of 450 nm within 20 min. Each experiment was conducted three times.

### Dual-luciferase reporter gene assay

The online website microRNA.org was applied to predict the binding site of miR-153 and LEPR. Dual luciferase reporter gene assay was carried out in order to verify that LEPR was the direct target of miR-153. The synthetic LEPR 3′UTR gene fragment was introduced into pMIR-reporter (Beijing Huayueyang Biotechnology Co., Ltd., Beijing, China) with the use of the endonuclease sites SpeI and HindIII, and the complementary sequence mutation sites of the seed sequences were designed on the LEPR wild type (WT). The target fragment was inserted into the pMIR-reporter plasmid with the use of T4 DNA ligase after the restriction enzymes. The correctly identified sequences fluorescent enzyme reporter plasmids WT, MUT were transfected respectively to the HEK-293T cell (Shanghai Beinuo Biotechnology Co., Ltd., Shanghai, China). After 48-h transfection, the cells were collected and split. The activity of luciferase was measured using the luciferase kit.

### Cell culture

The hippocampal tissue of the mice from the NS group and the VPA group were taken and added with 20% fetal bovine serum (FBS) at 37°C, which was gently percussed into suspension with the long glass pipette. Subsequently, the above mixture was filtered by a sterile 200-mesh stainless steel screen, after which centrifugation was carried out at 178 × ***g*** for 5 min to cultivate and re-suspend. After calculation, the cell suspension (2 × 10^6^ cells/ml) was inoculated into sterile culture bottles. The original medium was replaced by the complete medium after 24 h. After 72 h, the medium was added with cytarabine solution (TCI-C2035, Shanghai Spectrum Chemical Co., Ltd., Shanghai, China) to reach the final concentration of 2.5 mg/l, and replaced with a half volume. The culture medium was replaced every 3 d. The hippocampal neurons, which were cultured for 6 d, were transferred to the 96-well plate with 1× 10^4^ cells in each well. After 24-h culture, the cells were adhered and added with Aβ1-42 [β-amyloid peptide (1-42), Shanghai Moxi Biotech Co., Ltd, Shanghai, China] for 24-h incubation.

### Cell grouping and transfection

The cells collected from the VPA groups were sub-assigned into the following groups: the blank group (cell from the VPA group without any sequences), negative control (NC, cell from the VPA group transfected with miR-153 NC sequence), miR-153 mimic (cell from the VPA group transfected with miR-153 mimic), miR-153 inhibitor (cell from the VPA group transfected with miR-153 inhibitor), AG490 (cell from the VPA group, AG490 is the inhibitor of JAK-STAT signaling pathway, Cayman Chemical Company, Ann Arbor, Michigan, US, pretreating for 16 h) and miR-153 inhibitor + AG490 (cell from the VPA group transfected with miR-153 inhibitor and AG490). Besides, cells from the NS group were assigned into the normal group. All of the sequences were purchased from Gene Pharma Co., Ltd. (Shanghai, China). Prior to transfection, the cells were seeded in a six-well plate for 24 h. When cell confluence reached about 50%, the mice hippocampal neurons were transfected transiently under the mediation of the liposome lipofectamine2000 (11668027, Invitrogen, U.S.A.). The serum-free medium Opti-MEM (31985-070, Gibco, U.S.A.) (250 µl) was used to respectively dilute 10 µl liposome lipofectamine2000 as liquid A, and plasmids in each group with final concentration of 50 nM as liquid B. The liquid A and liquid B were mixed gently and incubated at room temperature for 5 min. Subsequently, the above two liquids were mixed and incubated at room temperature for 20 min and were added to cell culture well. Following incubation with 5% CO_2_ for 6–8 h at 37°C, the original medium was replaced by the complete medium and the cells were incubated for 24–28 h to conduct the following experiments.

### Reverse transcription quantitative polymerase chain reaction

Tissues (100 mg) were grinded to the uniform powder in liquid nitrogen, and were placed at room temperature for 5 min. The miRNeasy Mini Kit (217004, QIAGEN, Germany) was used to extract total RNA of tissue and cells. The primers of miR-153, LEPR, BDNF, BAX, Bcl-2, JAK, and STAT were designed and synthesized by TaKaRa (Takara Biotechnology Co., Ltd., Dalian, China) (Table 1). Next, RNA was reversely transcribed to cDNA with the use of the PrimeScript RT Kit (RR036A, Takara Biotechnology Ltd., Dalian, China) in the reverse transcription (RT) system (10 μl). The experiment was conducted in accordance with the instructions. The reaction liquid was used to conduct the fluorescent quantitative polymerase chain reaction (qPCR) according to the instructions of the SYBR® Premix Ex Taq^TM^ II Kit (RR820A, Takara Biotechnology Ltd., Dalian, China). ABI7500 quantitative PCR instrument (7500, ABI Company, Oyster Bay, NY, U.S.A.) was used for real-time fluorescence quantitative PCR detection. The relative expression of BDNF, Bcl-2 associated protein X (Bax), B cell lymphoma 2 (Bcl-2), JAK, and STAT was presented by using glyceraldehyde-3-phosphate dehydrogenase (GAPDH) as the internal reference and the relative expression of miR-153 was presented with U6 considered as the internal reference. The relative quantitative method and 2^−△Ct^ method were used to calculate the relative transcription level of target gene: △Ct = Ct_target gene_ − Ct_internal reference._ 2^−△Ct^ indicated the mRNA relative transcription expression of target gene. Each experiment was repeated three times.

**Table 1:** Primer sequences of miR-153, LEPR, BDNF, Bax, bcl-2, JAK, STAT, GAPDH, and U6 used for RT-qPCR

Gene	Primer sequences
miR-153	Forward 5-TCATTTTT GTGACGTTGCAG-3′
	Reverse 5-TGACTATGCAACTGGGCTCAT-3′
LEPR	Forward 5′-GGGACGATGTTCCAAACCCCA-3′
	Reverse 5′-AGGCTCCAGAAGAAGAGGACC-3′
BDNF	Forward 5′-GATGAGGACCAGAAGGTTGG-3′
	Reverse 5′-TGGGTAGTTGGGCATTG-3′
Bax	Forward 5′-GGATGCGTCCACCAAGAA-3′
	Reverse 5′-GGAGGAAGTCCAGTGTCC-3′
Bcl-2	Forward 5′-GACAGAAGATCATGCCGTCC-3′
	Reverse 5′-GGTACCAATGGCACTTCAAG-3′
JAK	Forward 5′-CTCTGACGTCTGGTCTTTTGG-3′
	Reverse 5′-GTTGGGCCTATCATTTTCAGGAAC-3′
STAT	Forward 5′-ACCTCCAGGACGCTTTGAT-3′
	Reverse 5′-TGTCTTCTGCACGTACTCCA-3′
GAPDH	Forward 5′-TGCCCCCATGTTTGTGATG-3′
	Reverse 5′-TGTGGTCATGAGCCCTTCC-3′
U6	Forward 5′-TCCGACGCCGCCATCTCTA-3′
	Reverse 5′-TATCGCACATTAAGCCTCTA-3′

Abbreviations: Bax, Bcl-2 associated protein X; Bcl-2, B cell lymphoma 2; BDNF, brain-derived neurotrophic factor; GAPDH, glyceraldehyde-3-phosphate dehydrogenase; JAK, janus kinase; LEPR, leptin receptor; miR-153, microRNA-153; RT-qPCR, reverse transcription quantitative polymerase chain reaction; STAT, signal transducers and activators of transcription; TGF-β1, transforming growth factor-β1.

### Western blot analysis

Total protein extract RIPA kit (R0010, Beijing Solarbio Science & Technology Co., Ltd., Beijing, China) was used to extract the total protein of fresh tissues and cells, and the BCA kit (20201ES76, Yeasen Biotech Co., Ltd., Shanghai, China) to determine the protein concentration of each sample. The samples were quantified according to different concentrations, and the protein was separated by electrophoresis with polyacrylamide gel. Subsequently, the protein was transferred to PVDF membrane by wet transfer assay and sealed with 5% bovine serum albumin for 1 h at room temperature. Subsequently, the membrane was added with diluted primary antibodies of rabbit anti-mice LEPR (1 : 2000, ab222823), BDNF (1 : 2000, ab108319), Bcl-2 (1 : 2000, ab59348), Bax (1 : 2000, ab53154), JAK (1 : 500, ab47435), STAT (1 : 2000, ab194898), p-JAK (1 : 1000-1 : 10000, ab32101), p-STAT (1 : 500-1 : 1000, ab194518), PI3K (1 : 1000, ab151549), p-Akt (1 : 500, ab38449) and Akt (1 : 10000, ab179463) overnight at 4°C. All of the above primary antibodies were purchased from Abcam Inc., Cambridge, MA, U.S.A. The membrane was washed with PBS five times (each time for 5 min) and was incubated with secondary antibody horseradish peroxidase labeled goat anti-rabbit antibody Immunoglobulin G (IgG) (1 : 5000, Beijing Zhongshan Biotech Co., Ltd., Beijing, China). The membrane was immersed in enhanced chemiluminescence (ECL) luminescent liquid (WBKLS0500, Pierce, Rockford, IL, U.S.A.), and the results were observed and photographed after the development in the dark room. The Bio-Rad image analysis system (Bio-Rad Laboratories, Hercules, CA, U.S.A.) was used to photograph the results and the Quantity One v4.6.2 software was used for analysis. Each experiment was repeated three times.

### 3-[4,5-Dimethylthiazol-2-yl]-2,5 diphenyl tetrazolium bromide assay

After a 48-h transfection, the cells were collected, counted, and inoculated in a 96-well plate with the density of 3 × 10^3^ to 6 × 10^3^ cells per well. The volume of each well was 0.1 ml, and six replicates were made. Three points of time were set: 24, 48, and 72 h. Each well was added with 20 μl 3-[4,5-dimethylthiazol-2-yl]-2,5 diphenyl tetrazolium bromide (MTT) solution (5 mg/ml) for incubation incubate at 37°C for 4 h, after which the culture was terminated. The culture supernatant was absorbed and abandoned, and each well was added with 150 μl dimethyl sulfoxide (DMSO) solution. The OD value of each well was read at 570 nm by using the enzyme-linked immunosorbent detector (NYW-96M, Beijing Nyaw Instruments Co., Ltd., Beijing, China). Each experiment was repeated three times. A curve chart of cell viability was obtained with time as the abscissa and OD value as the ordinate.

### Flow cytometry

After transfection for 48 h, the cells were collected and washed with cold PBS three times. Next, centrifugation was carried out with supernatant discarded and re-suspended with PBS. The cell concentration was adjusted to 1 × 10^5^ cells/ml. The cells were fixed by pre-cooled (−20°C) 75% ethanol (1 ml) at 4°C for 1 h, and centrifuged with cold ethanol, which was discarded afterwards. The cells were washed with PBS twice with the supernatant discarded, and added with 100 μl RNase A with the avoidance of light, and bathed at 37°C for 30 min. Cells were stained with 400 μl propidium iodide (PI) (40710ES03, Shanghai qcbio Science & Technologies Co., Ltd., Shanghai, China), which was mixed and incubated at 4°C for 30 min under dark conditions. Flow cytometer (653154, Shanghai Hengfei Science & Technologies Co., Ltd., Shanghai, China) was used to record red fluorescence at the excitation wavelength of 488 nm to detect cell cycle. The experiment was repeated three times.

After transfection for 48 h, the cells were detached by ethylenediaminetetraacetic acid (EDTA)-free trypsin, collected in flow tube, and centrifuged with the supernatant discarded. Subsequently, the cells were washed with cold PBS three times, and centrifuged with supernatant discarded. According to the instructions of Annexin-V-fluorescein isothiocyante (FITC) cell apoptosis detection kit (40302ES20, Shanghai qcbio Science & Technologies Co., Ltd., Shanghai, China), the Annexin-V-FITC/PI dye liquor was made in accordance to the proportion of 1 : 2 : 50 among Annexin-V-FITC, PI, and 2-[4-(2-hydroxyethyl)-1-piperazinyl] ethanesulfonic acid (HEPES) buffer solution. The cells (1 × 10^6^) were re-suspended per 100 μl dye liquor, mixed gently, and incubated at room temperature for 15 min. Subsequently, 1 ml HEPES buffer solution was added and mixed. Flow cytometer was promptly used for analysis and to detect the samples with 10^4^ cells which obtained at each time. Data were analyzed through Cell Quest software (Becton, Dickinson and Company, NJ, U.S.A.) in order to detect apoptosis. The experiment was repeated three times.

### Statistical analysis

All data were analyzed by using SPSS 21.0 (IBM Corp, Armonk, NY, U.S.A.). Measurement data were expressed as mean ± standard deviation. Comparisons between two groups were performed by *t* test, while comparisons among multiple groups were conducted by one-way analysis of variance (ANOVA). *P* < 0.05 was considered statistically significant.

## Results

### The autistic mice models were successfully established

The developmental condition of mice in two groups was observed in order to assess the successful establishment of the mice models. The comparison of the development of mice in the VPA and NS groups is shown in Table 2. There was no significant difference in body weight between mice in the VPA and NS groups at P7 and P14 (*P* > 0.05), while the VPA mice had markedly lower weight than the NS mice at P21, P56, and P90 (*P* < 0.05). The eye opening time was observed and recorded. At P12, mice in the VPA and NS groups did not open eyes. At P13, some of mice in the NS group opened eyes while the mice in the VPA group still had their eyes closed. At P14 and P15, more of the mice in the NS group had their eyes open compared with that of the VPA group. The differences among P12, P13, P14, and P15 were statistically significant (*P* < 0.05). At P16, all the mice in the two groups had their eyes completely open without any significant difference (*P* > 0.05). Compared with the NS group, mice in the VPA group used more time in complete turning, and the differences in time among P7, P8, P9, and P10 were statistically significant (*P*<0.05). Compared with the NS group, mice in the VPA group were less capable of swimming and their coordination reaction was poor. The difference in time among P9, P11, and P13 was statistically significant (*P* < 0.05), and there was no significant difference between the two groups at P15 (*P* > 0.05). The results showed that the model of autistic mice was successfully established.

**Table 2:** Comparison of weight, eye opening time evaluation, turning tendency, and swimming ability of autistic mice in the NS and VPA groups

	Cases	NS	VPA	*P* value
Weight (g)				
P7 d	10	6.60 ± 1.51	6.20 ± 1.40	0.547
P14 d	10	8.70 ± 1.64	8.50 ± 1.51	0.78
P21 d	10	12.50 ± 0.97	10.20 ± 1.03	<0.001
P56 d	10	30.70 ± 3.37	25.10 ± 1.85	<0.001
P90 d	10	53.40 ± 5.21	40.60 ± 6.60	<0.001
Eye opening time evaluation (point)				
P12 d	10	0.00 ± 0.00	0.00 ± 0.00	/
P13 d	10	0.70 ± 0.48	0.00 ± 0.00	0.026
P14 d	10	1.10 ± 0.74	0.80 ± 0.42	0.039
P15 d	10	1.60 ± 0.52	1.10 ± 0.57	0.038
P16 d	10	2.00 ± 0.16	2.00 ± 0.33	/
Turning tendency (s)				
P7 d	10	17.70 ± 2.50	29.40 ± 3.78	<0.001
P8 d	10	15.70 ± 1.42	21.50 ± 2.22	<0.001
P9 d	10	8.60 ± 1.35	14.60 ± 1.07	<0.001
P10 d	10	6.70 ± 1.06	11.20 ± 0.63	<0.001
Swimming ability (point)				
P9 d	10	1.80 ± 0.92	1.20 ± 0.79	0.018
P11 d	10	2.40 ± 0.84	1.90 ± 0.57	0.035
P13 d	10	2.70 ± 0.48	2.40 ± 0.52	0.004
P15 d	10	3.40 ± 0.70	2.80 ± 0.63	0.059

Abbreviations: NS, normal saline; VPA, sodium valproate.

### The memory ability of the mice after treatment with VPA was evidently impaired

The escape latency was applied to determine the space learning and memory ability, and therefore, Morris water maze (MWM) was conducted in order to test the space learning and memory ability of mice in the two groups. The experimental results showed that the escape latency of the NS and VPA groups from the first day to the fifth day had a decreasing trend with the increasing days, indicating that mice had certain learning ability in the five-day training. Compared with the NS group, mice in the VPA group had significantly prolonged escape latency (*P* < 0.05) (Table 3). The results showed that there was a significant damage in the memory damage of mice in the VPA group. The model establishment was successful, with a success rate of 100%.

**Table 3:** Changes in the escape latency (s) of autistic mice in the NS and VPA groups

	NS group	VPA group	*P* value
The 1st day	59.60 ± 10.31	96.30 ± 10.91	< 0.001
The 2nd day	31.40 ± 6.04	84.80 ± 11.20	< 0.001
The 3rd day	28.40 ± 3.66	70.70 ± 8.23	< 0.001
The 4th day	18.70 ± 3.37	51.70 ± 5.38	< 0.001
The 5th day	13.20 ± 2.44	34.50 ± 4.20	< 0.001

Abbreviations: NS, normal saline; VPA, sodium valproate.

### BDNF is down-regulated and LEPR is up-regulated in mice with autism

**Figure 1:**
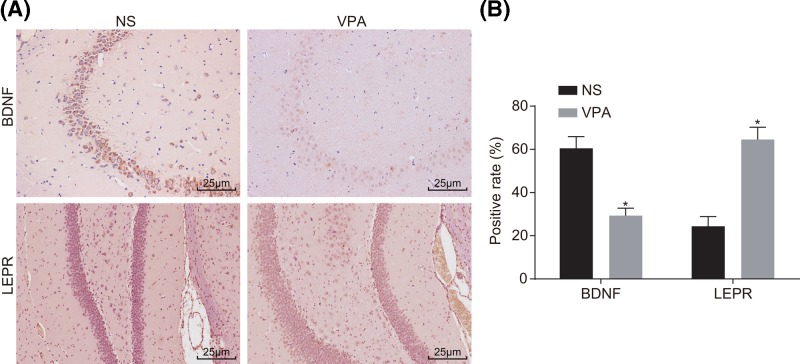
Immunohistochemical staining (×400) reveals decreased expression of BDNF and increased expression of LEPR in autistic hippocampal neuron cells (**A**) Positive expression results of BDNF and LEPR in the NS and VPA groups; (**B**) quantitative analysis for positive rate of BDNF and LEPR protein expression in the NS and VPA groups; *, *P* < 0.05 compared with the NS group. Abbreviations: BDNF, brain-derived neurotrophic factor; LEPR, leptin receptor; NS, normal saline; VPA, sodium valproate.

The results of immunohistochemical staining showed that the positive expression of BDNF was mainly distributed in the cytoplasm and membrane of the nerve cells with brown and yellow color (Figure 1A). The positive expression rate of BDNF protein in the VPA group was (29.32 ± 3.45)%, which was significantly lower than that in the NS group [(64.59 ± 5.64)%]. The positive expression rate of LEPR protein was (60.49 ± 5.41)% in the VPA group, and (24.36 ± 4.55)% in the NS group. Compared with the NS group, the VPA group exhibited significantly increased positive expression rate of LEPR and decreased expression rate of BDNF (*P* < 0.05) (Figure 1B). These findings suggested that autistic mice had decreased BDNF expression and increased LEPR increases.

### The apoptotic rate in the VPA group is higher

**Figure 2:**
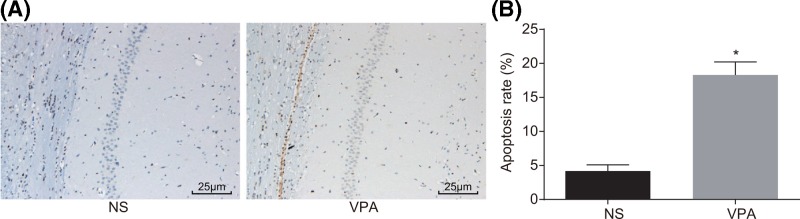
TUNEL staining (×400) demonstrates a higher apoptosis rate in mice with autism (**A**) TUNEL staining results of mice in the NS and VPA groups; (**B**) comparison of apoptosis rate between the NS group and the VPA group; *, *P* < 0.05 compared with the NS group; TUNEL, transferase-mediated deoxyuridine triphosphate-biotin nick end labeling. Abbreviations: NS, normal saline; VPA, sodium valproate.

Hippocampal neuron cell apoptosis was observed after TUNEL staining, and the result showed that there were a large number of brown apoptotic cells in the VPA group (Figure 2A), and the apoptotic rate in the NS group was significantly lower than that in VPA tissues (*P* < 0.05) (Figure 2B). Based on this finding, a conclusion can be drawn that hippocampal neuron cell apoptosis is promoted in mice with autism.

### The expressions of IL-6, IL-1β, and TNF-α in autistic mice increased significantly

ELISA was performed in order to investigate the inflammatory factor levels, and the results in Table 4 showed that compared with the NS group, the VPA group significantly increased the expression of IL-6, IL-1β, and TNF-α in the serum (*P* < 0.05). These observations indicated that inflammatory reaction is enhanced in mice with autism.

**Table 4:** Serum levels of TNF-α, IL-6, and IL-1β in autistic mice in the NS and VPA groups

	NS	VPA	*P* value
TNF-α (ng/ml)	0.71 ± 0.08	1.36 ± 0.15	<0.001
IL-6 (pg/ml)	94.36 ± 8.51	178.90 ± 17.94	<0.001
IL-1β (pg/ml)	5.19 ± 0.54	8.43 ± 0.88	<0.001

Abbreviations: IL, interleukin; NS, normal saline; TNF-α, tumor necrosis factor alpha; VPA, sodium valproate.

### Up-regulated miR-153, BDNF, and Bcl-2 as well as down-regulated LEPR, Bax, JAK, and STAT were found in brain tissues

**Figure 3:**
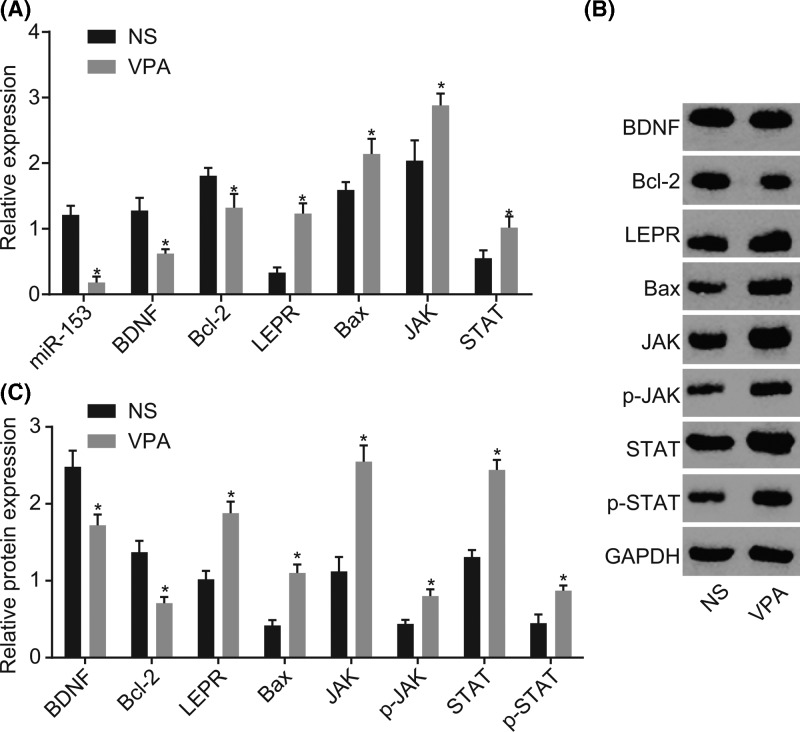
In vivo experiment focused on expression of miR-153, LEPR, Bax, JAK, STAT, BDNF, and Bcl-2 in brain tissues, and decreased miR-153, BDNF, and Bcl-2 while increased LEPR, Bax, JAK, and STAT as well as p-JAK and p-STAT were found (**A**) RT-qPCR was applied for detecting miR-153 expression and mRNA expression of LEPR, Bax, JAK, STAT, BDNF, and Bcl-2; (**B**) Western blot analysis for the protein expression of BDNF, Bcl-2, LEPR, Bax, JAK, p-JAK, STAT, and p-STAT between the NS group and the VPA group; (**C**) gray value analysis of BDNF, bcl-2, LEPR, Bax, JAK, p-JAK, STAT, and p-STAT with GAPDH as the internal reference; *, *P* < 0.05 compared with the NS group. Abbreviations: Bax, Bcl-2 associated protein X; Bcl-2, B cell lymphoma 2; BDNF, brain-derived neurotrophic factor; GAPDH, glyceraldehyde-3-phosphate dehydrogenase; JAK, janus kinase; LEPR, leptin receptor; miR-153, microRNA-153; NS, normal saline; RT-qPCR, reverse transcription-quantitative polymerase chain reaction; STAT, signal transducers and activators of transcription; VPA, sodium valproate.

RT-qPCR and Western blot analysis were used to detect the expressions of miR-153, LEPR, BDNF, Bax, Bcl-2, JAK, and STAT. As shown in Figure 3, the VPA group presented with significantly reduced miR-153, mRNA, and protein expression of BDNF and Bcl-2, and increased mRNA and protein expression of LEPR, Bax, JAK, and STAT as well as p-JAK and p-STAT in comparison with the NS group (*P* < 0.05). The aforementioned results suggest that there is a poor expression of miR-153 and high expression of LEPR in brain tissues in the autistic mice with the activation of the JAK-STAT signaling pathway.

### LEPR is a target gene of miR-153

**Figure 4:**
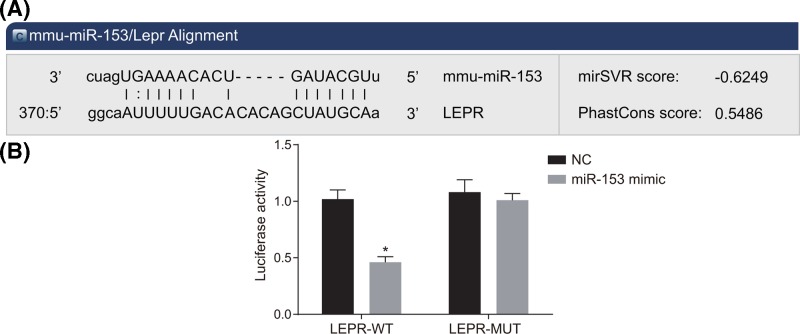
LEPR is confirmed as a target of miR-153 using dual-luciferase reporter assay (**A**) The target relationship of website prediction between miR-153 and LEPR; (**B**) dual-luciferase report: the luciferase activity of LEPR-Wt and LEPR-3′UTR between the NC and miR-153 mimic group; *, *P* < 0.05 compared with the NS group. Abbreviations: LEPR, leptin receptor; miR-153, microRNA-153; NC, negative control; NS, normal saline; UTR, untranslated region; VPA, sodium valproate; WT, wild-type.

The bioinformatics prediction website (http://www.microRNA.org) was used to analyze target gene of miR-153, and dual luciferase reporter gene assay was applied to verify the target relationship between miR-153 and LEPR (Figure 4). The experimental results revealed that compared with the NC group, the co-transfection of miR-153 mimic and Wt-LEPR group resulted in the reduction in the luciferase signal (*P* < 0.05), while there was no significant difference in MUT 3′UTR (*P* > 0.05), indicating that miR-153 can specifically bind to the LEPR gene.

### Up-regulated miR-153 inhibits LEPR and JAK-STAT signaling pathway

**Figure 5:**
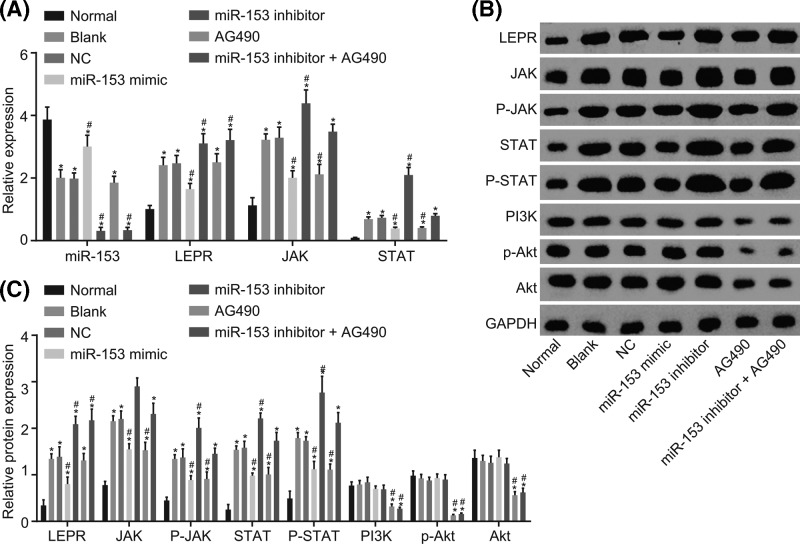
RT-qPCR and Western blot analysis reveal that overexpressed miR-153 inhibits LEPR and the JAK-STAT signaling pathway (**A**) RT-qPCR was applied for detecting miR-153 expression and mRNA expression of LEPR, JAK, STAT, PI3K, and Akt in cells after transfection; (**B**) Western blot analysis for the protein expression of LEPR, JAK, p-JAK, STAT, p-STAT, PI3K, Akt, and p-Akt in cells after transfection; (**C**) gray value analysis of LEPR, JAK, p-JAK, STAT, p-STAT, PI3K, Akt, and p-Akt with GAPDH as the internal reference; *, *P* < 0.05 compared with the normal group; #, *P* < 0.05 compared with the blank and NC groups. Abbreviations: GAPDH, glyceraldehyde-3-phosphate dehydrogenase; JAK, janus kinase; LEPR, leptin receptor; miR-153, microRNA-153; NC, negative control; PI3K, phosphatidylinositol 3′-kinase; RT-qPCR, reverse transcription-quantitative polymerase chain reaction; STAT, signal transducers and activators of transcription.

RT-qPCR and Western blot analysis were performed to detect the expression of miR-153, LEPR, JAK, and STAT in order to investigate the mechanisms and functions of miR-153 in autism. Compared with the normal group, the blank, NC, miR-153 mimic, miR-153 inhibitor, AG490, and miR-153 inhibitor + AG490 groups had down-regulated miR-153 expression, and increased mRNA and protein expression of LEPR, JAK, and STAT as well as p-JAK and p-STAT (*P* < 0.05). There was no significant difference between the blank and NC groups (*P* > 0.05). Compared with the blank and NC groups, the miR-153 mimic group had higher expression of miR-153 and lower LEFR expression, additionally, the mRNA and protein expression of JAK and STAT as well as p-JAK and p-STAT was down-regulated (*P* < 0.05); however, there was no evident difference in the expression of miR-153 and LEPR in the AG490 group while the expression of JAK, STAT, p-JAK, and p-STAT was down-regulated (*P* < 0.05); the expression of miR-153, and mRNA and protein expression of LEPR, JAK, and STAT as well as p-JAK and p-STAT were up-regulated in the miR-153 inhibitor group (*P* < 0.05). The miR-153 inhibitor + AG490 group had down-regulated expression of miR-153 and up-regulated expression of LEPR while the other factors presented with no evident difference (*P* > 0.05). Moreover, there was a significant decreased in the expression of PI3K, p-Akt, and Akt in the AG490 and miR-153 inhibitor + AG490 groups compared with the normal group (*P* < 0.05) while there was no significant difference observed in the remaining groups (*P* > 0.05) (Figure 5). These findings highly suggested that miR-153 results in the inhibition of JAK-STAT signaling pathway activation through the down-regulation of LEPR and has no significant effect on the activation of the PI3K-Akt signaling pathway.

### Up-regulated miR-153 promotes cell proliferation

**Figure 6:**
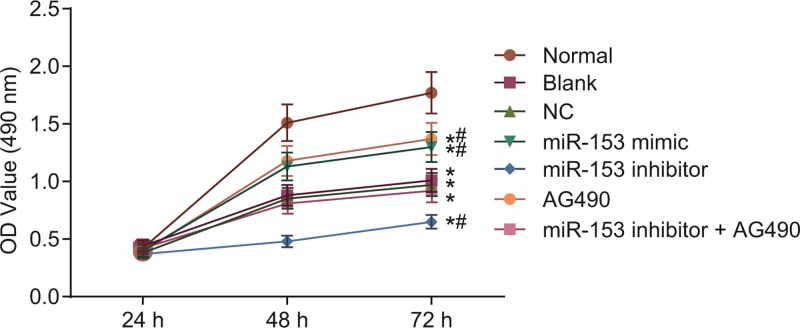
The MTT assay shows that overexpressed miR-153 promotes cell viability *, *P* < 0.05 compared with the normal group at 48 and 72 h; #, *P* < 0.05 compared with the blank and NC groups at 48 and 72 h. Abbreviations: miR-153, microRNA-153; MTT, 3-(4,5-dimethyl-2-thiazolyl)-2,5-diphenyl-2-H-tetrazolium bromide; NC, negative control.

In the following experiments, the effect of miR-153 on cell biological processes was investigated. MTT assay was conducted to examine cell viability, and as shown in Figure 6, the results showed that there was no significant difference in cell proliferation in each group at 24 h (*P* > 0.05). Compared with the normal group, the blank, NC, miR-153 mimic, miR-153 inhibitor, AG490, and miR-153 inhibitor + AG490 groups had decreased cell proliferation (*P* < 0.05). Compared with the blank group, there was no significant difference in the NC group (*P* > 0.05). Compared with the blank and NC groups, the miR-153 mimic and AG490 groups had increased cell proliferation, and the miR-153 inhibitor group had decreased cell proliferation (*P* < 0.05). There was no significant difference in cell proliferation among the miR-153 inhibitor + AG490 group, the blank group, and the NC group (*P* > 0.05). Based on the above findings, it can be concluded that up-regulation of miR-153 leads to the enhancement in the proliferative ability of cells in autism.

### Up-regulation of miR-153 blocks cell cycle progression

**Figure 7:**
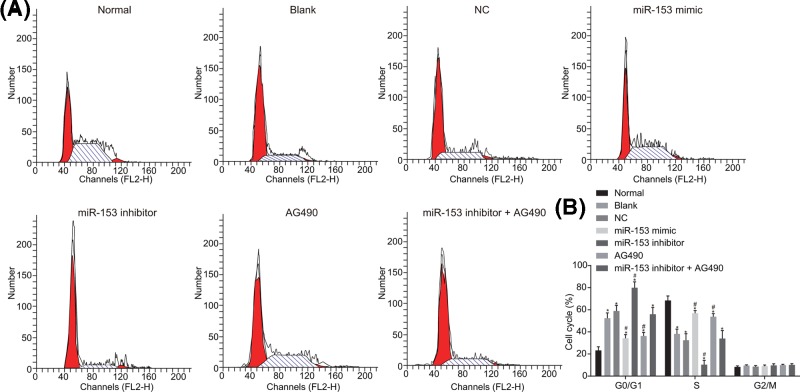
Flow cytometric data demonstrate that overexpression of miR-153 arrested cells in G1 phase (**A**) Flow cytometry of cells after different transfection; (**B**) the cell cycle changed after transfection; *, *P* < 0.05 compared with the normal group; #, *P* < 0.05 compared with the blank and NC groups. Abbreviations: miR-153, microRNA-153; NC, negative control.

The flow cytometry assay was conducted to examine the cell cycle, and the results (Figure 7) revealed that compared with the normal group, the blank, NC, miR-153 mimic, miR-153 inhibitor, AG490, and miR-153 inhibitor + AG490 groups had increased cells in G1 phase while decreased cells in S phase (all *P* < 0.05). There was no significant difference in cell cycle between the blank and NC groups (*P* > 0.05). Compared with the blank and NC groups, the miR-153 mimic and AG490 groups had decreased proportion of cells in G1 phase, and increased in S phase, while the miR-153 inhibitor group had increased cells in G1 phase and decreased cells in S phase (*P* < 0.05); there was no significant difference in cell cycle of the miR-153 inhibitor + AG490 group (*P* > 0.05). Therefore, miR-153 is involved in cell cycle distribution in autism.

### Up-regulation of miR-153 inhibits cell apoptosis

**Figure 8:**
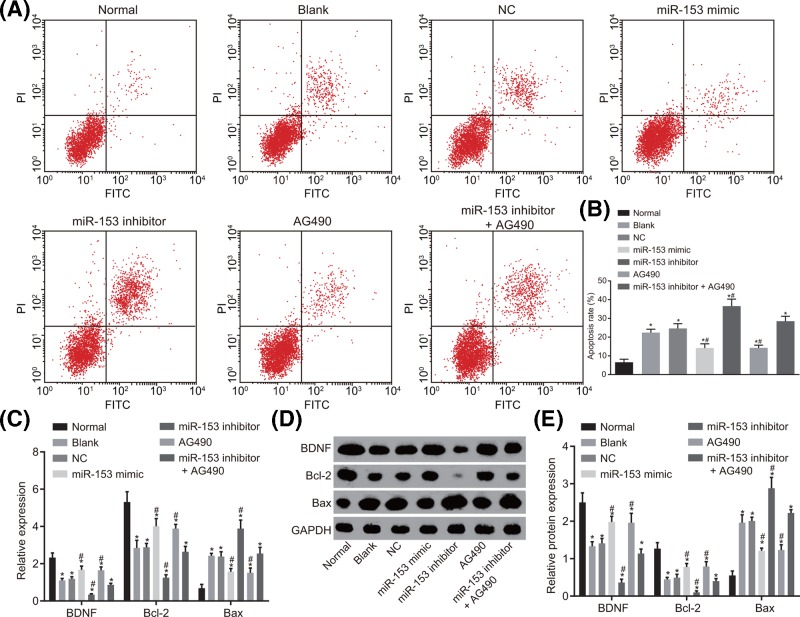
Flow cytometric data demonstrate that the cell apoptosis was inhibited after transfection of overexpressed miR-153 (**A**) Flow cytometry for cell apoptosis after transfection; (**B**) apoptosis rate after transfected with different factors; (**C**) mRNA expression of apoptosis-related factors (BDNF, Bcl-2, and Bax) determined by RT-qPCR; (**D,E**) relative protein expression of apoptosis-related factors (BDNF, Bcl-2, and Bax) normalized to GAPDH determined by Western blot analysis; *, *P* < 0.05 compared with the normal group; #, *P* < 0.05 compared with the blank and NC groups. Abbreviations: Bax, Bcl-2 associated protein X; Bcl-2, B cell lymphoma 2; BDNF, brain-derived neurotrophic factor; GAPDH, glyceraldehyde-3-phosphate dehydrogenase; miR-153, microRNA-153; NC, negative control; RT-qPCR, reverse transcription-quantitative polymerase chain reaction.

Flow cytometry assay was performed in order to examine cell apoptosis in combination with RT-qPCR and Western blot analysis in order to find supporting evidence regarding the expression levels of apoptosis-related factors (BDNF, Bcl-2, and Bax). Based on the results shown in Figure 8, compared with the normal group, the blank, NC, miR-153 mimic, miR-153 inhibitor, AG490, and miR-153 inhibitor + AG490 groups had significantly increased apoptotic rate (*P* < 0.05), which was further approved by the lower levels of BDNF and Bcl-2 along with higher Bax level (*P* < 0.05). The difference with regard to cell apoptosis rate and expression levels of apoptosis-related factors (BDNF, Bcl-2, and Bax) between the blank group and the NC group was not statistically significant (*P* > 0.05). Compared with the blank and NC groups, the miR-153 mimic group and the AG490 group had decreased apoptotic rate with up-regulated BDNF and Bcl-2 and down-regulated Bax, while the miR-153 inhibitor + AG490 group had opposite changing tendency (*P* < 0.05). There was no significant difference of cell apoptosis or the expression levels of apoptosis-related factors (BDNF, Bcl-2, and Bax) among the miR-153 inhibitor + AG490 group, the blank group and the NC group (*P* > 0.05). These findings were highly indicative of the inhibitory effects exerted by up-regulated miR-153 on cell apoptosis.

## Discussion

Autism is a persistent neurodevelopmental impairment which has a high morbidity of 0.88%, which is substantially higher than the previously recognized estimate [1]. The overexpression of miR-153 leads to the obvious decrease in the expression of alpha-synuclein, and its involvement in Parkinson disease has been studied and proven [14]. Due to the multiple potential targets that exist for each miRNA, one challenge in understanding functional miRNA contributions in BDNF expression and apoptosis of hippocampal neurons has been the identification of these targets. The present study was conducted with the main focus of understanding the mechanism by which biological function of miR-153 affects autism. Consequently, the present study demonstrates that miR-153 blocks JAK-STAT signaling pathway through the inhibition of LEPR, thus regulating BDNF expression and proliferation of hippocampal neurons.

Initially, the learning and memory abilities, BDNF and LEPR expression, as well as inflammatory reaction were evaluated. In line with our study, Gao et al. also demonstrated in their study that prenatal exposure to VPA in newborn rat could induce experimental autism with neurobehavioral aberrations which presented as dysfunctions in learning and memory as well as increased apoptosis of hippocampus [22]. BDNF, a well-known neurotrophin fundamental for brain development and function, has also been shown to be highly expressed in autism [23]. An interesting finding suggested that there is an up-regulation in circulating leptin in children suffering from different forms of neurodevelopmental disorders, one of which being autism spectrum disorders [24]. Moreover, there are signs of neuro-inflammation, immune abnormalities as well as altered inflammatory responses observed in people suffering autism during their entire lifetime [25].

In the following, in vitro experiments were performed to investigate the effect of miR-153 on LEPR, the JAK-STAT signaling pathway, BDNF expression as well as proliferation and apoptosis of hippocampal neurons. The final result revealed that overexpression of miR-153 could result in the enhancement of BDNF expression and proliferation of hippocampal neurons by inhibiting the JAK-STAT signaling pathway and LEPR. Based on the target prediction program and the luciferase activity determination, we found that LEPR is a putative target gene of and negatively regulated by miR-153. Kim et al. found that miR-153 was down-regulated and correlated with advanced clinical stage in ovarian epithelial tumors, contributing to epithelial–mesenchymal transition and tumor metastasis in human epithelial cancer [26,27]. The up-regulation of miR-153 has also been found to promote cell proliferation by down-regulating the PTEN tumor suppressor gene in patients of prostate cancer, which we found during our intensive research of previous articles [28]. Fragkouli and Doxakis also suggested that miR-153 provides protection for neurons against death by down-regulating the mTOR signaling pathway [29], which was consistent with our study. Regulated by both genetic and environmental factors, BDNF is required for the enhancement of hippocampal neurogenesis [30]. The JAK-STAT signaling pathway has been proven to be necessary for the development and function of innate and adaptive immunity, and its abnormal activation was found to be involved in a number of neuro-inflammatory diseases [19]. Another important finding is that the activation of JAK2-STAT1 signaling pathway could mediate astrogliosis, which is in brains of scrapie-infected mice [31]. Leptin plays an important role in the modulation of the neuroendocrine system, brain development, reproductive system, bone development, as well as immune system [32]. LEPR is widely found in human brain, including the cortex, amygdala, hippocampus, and thalamus, with highest levels observed in hypothalamic nuclei [33]. LEPR has been specifically found to be widely distributed throughout the hippocampus, which plays a crucial role in learning and memory [24]. In addition, leptin is part of the same protein family as IL-6, an inflammatory cytokine, and its dysregulation has been correlated with psychopathology, and previous reports have demonstrated that this relationship arises from the inflammatory function of leptin [34–36]. Furthermore, Valleau et al. also pointed out that leptin could activate signal transducer and activator of transcription-3 signaling in agouti-related peptide neurons in the arcuate nucleus, promoting an increase in locomotor activity [24], which was partly in line with our study.

## Conclusions

The aforementioned findings led to the conclusion that miR-153 is down-regulated among autistic mice, in relation to autism progression. The present study also demonstrated that LEPR is a target gene of miR-153 in autism. The overexpression of miR-153 significantly reduces apoptosis and promotes proliferation in hippocampal neuron cells via the inhibition of the JAK-STAT signaling pathway through inhibiting LEPR gene. Thus, miR-153 may be a promising therapeutic target for autism.

## Abbreviations

Bax: Bcl-2 associated protein X
Bcl-2: B cell lymphoma 2
BDNF: brain-derived neurotrophic factor
DAB: diaminobenzidine
ELISA: enzyme-linked immunoassay
EO: eyes open
FITC: fluorescein isothiocyante
GAPDH: glyceraldehyde-3-phosphate dehydrogenase
HEPES: 2-[4-(2-hydroxyethyl)-1-piperazinyl] ethanesulfonic acid
IL-6: interleukin-6
JAK-STAT: janus kinase-signal transducer and activator of transcription
LEPR: leptin receptor
miRNAs: microRNAs
MTT: 3-[4,5-dimethylthiazol-2-yl]-2,5 diphenyl tetrazolium bromide
MWM: Morris water maze
OD: optical density
PBS: phosphate buffer solution
PI: propidium iodide
POD: peroxidase
RT-qPCR: reverse transcription quantitative polymerase chain reaction
TdT: terminal deoxynucleotidyl transferase
TNF-α: tumor necrosis factor alpha
TUNEL: terminal deoxynucleotidyl transferase (TdT)-mediated dUTP nick end labeling
WT: wild type
